# Cognitive Network Digital Twins: A Survey of Concepts, Architectures, and Future Research Directions

**DOI:** 10.3390/s26216766

**Published:** 2026-10-26

**Authors:** Abhik Banerjee, Md Mashiur Rahman, Asif Ahmed Sardar, Aravinda S. Rao, Jonathan Kua, Goutam Das, Prem Prakash Jayaraman

**Affiliations:** 1 School of Science, Computing and Emerging Technologies, Swinburne University of Technology, Melbourne, VIC 3122, Australia; abanerjee@swin.edu.au (A.B.); mdmashiurrahman@swin.edu.au (M.M.R.); 2 School of Electrical Engineering, Computing and Mathematical Sciences, Curtin University, Perth, WA 6102, Australia; asif.sardar@curtin.edu.au; 3 Department of Computer Science & Engineering, RNS Institute of Technology, Bengaluru 560098, India; aravindasrao@rnsit.ac.in; 4 School of Information Technology, Deakin University, Geelong, VIC 3220, Australia; jonathan.kua@deakin.edu.au; 5 Department of Electronics and Electrical Communication Engineering, Indian Institute of Technology Kharagpur, Kharagpur 721302, India; gdas@gssst.iitkgp.ac.in

**Keywords:** Cognitive Network Digital Twins, 5G/6G networks, Industry 5.0

## Abstract

The increasing adoption of data-intensive and real-time Artificial Intelligence (AI) and Internet of Things (IoT) applications in domains such as manufacturing, healthcare, smart cities, and remote mining has placed significant pressure on current 5G and emerging 6G networks, exposing the limitations of conventional reactive network management approaches. In recent years, Network Digital Twins (NDTs) have been proposed to address some of these challenges by creating real-time virtual replicas for monitoring and optimisation. Recent studies have increasingly incorporated learning-based optimisation and adaptive decision-making into Digital Twin-based network systems; however, these capabilities are often developed for specific optimisation or control tasks. In this paper, we define and formalise the concept of Cognitive Network Digital Twins (CNDTs), which extends traditional NDTs with cognitive capabilities including perception, learning, reasoning, prediction, and autonomous decision-making. In emerging data-intensive applications, for example, in Industry 5.0, these autonomous capabilities are intended to complement human expertise and interaction, allowing human operators to provide guidance or intervention where required. We review the state of the art in Digital Twins and intelligent network systems, trace the technological evolution that motivates CNDTs, and introduce a multi-layered architectural taxonomy that organises the key components supporting cognitive functions in CNDTs. We also examine the potential of CNDTs to improve network resilience, efficiency, and scalability and identify key research challenges related to cognitive intelligence, semantic knowledge management, architectural placement, and autonomous control. This work establishes a clear conceptual foundation for CNDTs and highlights their importance as an emerging framework for intelligent and self-adapting network management in future wireless systems.

## 1. Introduction

The rapid evolution of telecommunication networks, driven by advancements in 5G and the anticipated rollout of 6G, has led to increasingly complex, uncertain, and dynamic network environments [1,2]. These networks are expected to support ultra-low-latency and high-reliability services for applications such as industrial automation, smart cities, and remote healthcare [3,4]. However, traditional network management approaches struggle to cope with modern networked systems’ growing scale, complexity, and real-time decision-making requirements. While network management approaches have evolved over several decades, from protocol-based approaches with Simple Network Management Protocol (SNMP) standardisation (1990) [5] to the emergence of Software-Defined Networking (2008–2012) [6,7] that separate control and data planes, the transition towards 5G and 6G networks coupled with the growth of diverse data-intensive applications has necessitated a shift from reactive to proactive network management paradigms.

The Digital Twin (DT) concept was first introduced by Grieves in the context of manufacturing in 2002 [8] as a virtual representation that serves as a real-time digital counterpart of physical objects, processes, or systems. More recently, Cognitive Digital Twins (CDTs) have been introduced [9], incorporating AI capabilities to enable autonomous decision-making. While Network Digital Twins (NDTs) have been explored in the literature, drawing upon existing DT concepts [10], they have primarily been focused on network monitoring and management. However, the role of cognition remains unexplored within NDTs, especially in pursuit of proactive adaptation to an increasing range of application scenarios.

In this paper, we review the current state of the art in NDTs with regard to their cognitive capabilities in order to support emerging data-intensive applications in various industry domains. Subsequently, we introduce Cognitive Network Digital Twins (CNDTs) and their key functions. Finally, we outline some future research directions. The key contributions of the paper are as follows:

- A comprehensive review of the cognitive capabilities of existing Network Digital Twins (NDTs);
- Introduction of Cognitive Network Digital Twins (CNDTs), and a CNDT architecture presenting its key functions;
- A future research agenda for CNDTs.

The rest of the paper is structured as follows. In Section 2, we investigate use cases in three industrial domains to identify the key network functions required to support emerging data-intensive applications. Based on the requirements identified in Section 2, we study the existing landscape of Network Digital Twins (NDTs) in Section 3 and how they relate to cognitive capabilities for wireless networks. In Section 4, we first summarise the gaps in existing NDTs and then formally introduce Cognitive Network Digital Twins (CNDTs). Subsequently, we introduce a conceptual architecture for CNDTs, which organises the key CNDT functions into four layers. Given the functions identified in Section 4, Section 5 presents open challenges and future directions for CNDT research. Finally, Section 6 concludes the paper.

## 2. Motivation

We first identify the cognitive requirements of emerging 5G and 6G networks supporting data-intensive applications. We then examine the role of cognitive capabilities in shaping how networks operate. AI-driven decision-making, real-time adaptability, and predictive analytics have the potential to drive network efficiency by enabling proactive resource management, improved reliability, and enhanced security in complex network environments. We examine the network requirements for emerging applications in three domains, namely manufacturing, smart cities and mining.

### 2.1. Smart Manufacturing

In an Industry 5.0 system, the factory floor will integrate advanced technological systems to enable remote human interaction and autonomous operation. In such environments, autonomous capabilities are intended to support human operators rather than eliminate their involvement, with humans retaining the ability to monitor and intervene when required. It is expected that future factories will comprise a fleet of Autonomous Guided Vehicles (AGVs) and cameras for floor monitoring. Remote human workers will have access to the factory environment through Virtual Reality (VR) telepresence systems, allowing intervention and monitoring from a separate location [11,12], as well as in scenarios that involve a combination of these technologies [13]. During remote access events, workers will interact with mobile robotic assistants on the factory floor equipped with advanced sensing capabilities, including object detection and video streaming [14].

**Figure 1:**
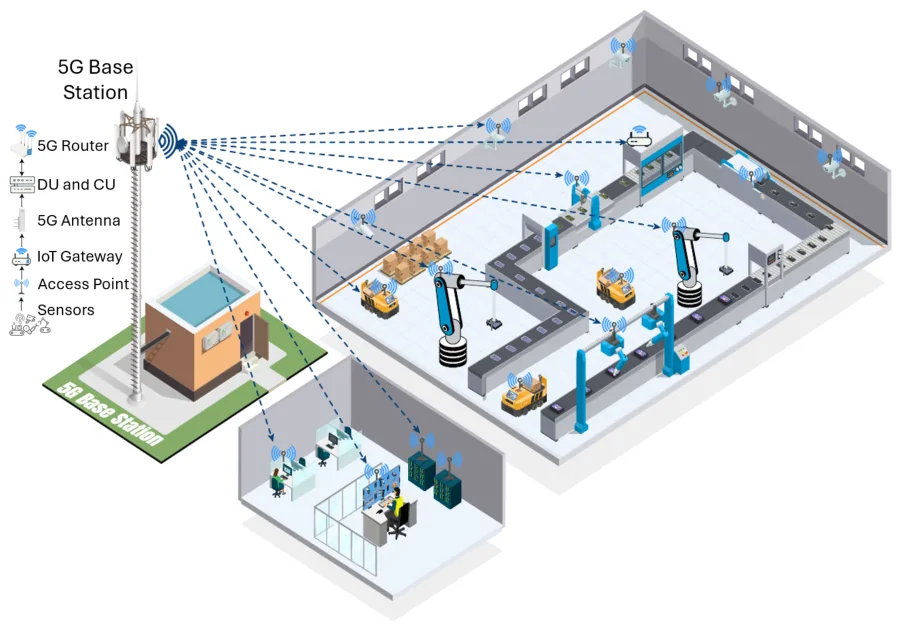
An illustration of a 5G-connected Industry 5.0 system.

In [15], the authors consider the network resource allocation problem in an Industry 5.0 setup capable of AR/VR-assisted remote human access. The authors show that making the resource allocator aware of the state of the industrial applications leads to efficient bandwidth usage. In addition, it increases the scalability of the industrial system. This resource allocation method reacts to changes in the states of the Industrial applications and has some inherent limitations. Industrial operations often involve processes distributed across multiple locations. In the event of a fault at one location on the factory floor, expert assistance via an AR/VR system may be necessary [16]. Network performance in industrial environments is impacted by various factors, including interference from industrial machinery, fluctuating network loads, and dynamic floor layouts that obstruct wireless signal propagation. These challenges make efficient resource allocation particularly difficult, as real-time applications such as robotic coordination, predictive maintenance, and Digital Twins demand ultra-reliable, low-latency communication. The unpredictability of network conditions further complicates the scheduling of time-sensitive tasks, leading to potential delays and inefficiencies in the Industry 5.0 ecosystem. A representative 5G-connected Industry 5.0 system for this operating context is shown in Figure 1.

Therefore, some of the key network functions required to support data-intensive Industry 5.0 applications include the following.

1. *Resource allocation based on application requirements:* Optimal resource allocation is crucial for supporting emerging applications in manufacturing environments. Especially in scenarios with multiple concurrent applications on the same network, there is a need for dynamic adaptation of network resources based on application requirements.
2. *Environment-based network condition monitoring:* As manufacturing environments are known to impact wireless network performance, ensuring reliable connectivity for high-bandwidth low-latency applications implies the need for applications to adapt based on dynamic network conditions.

### 2.2. Smart City Applications

**Figure 2:**
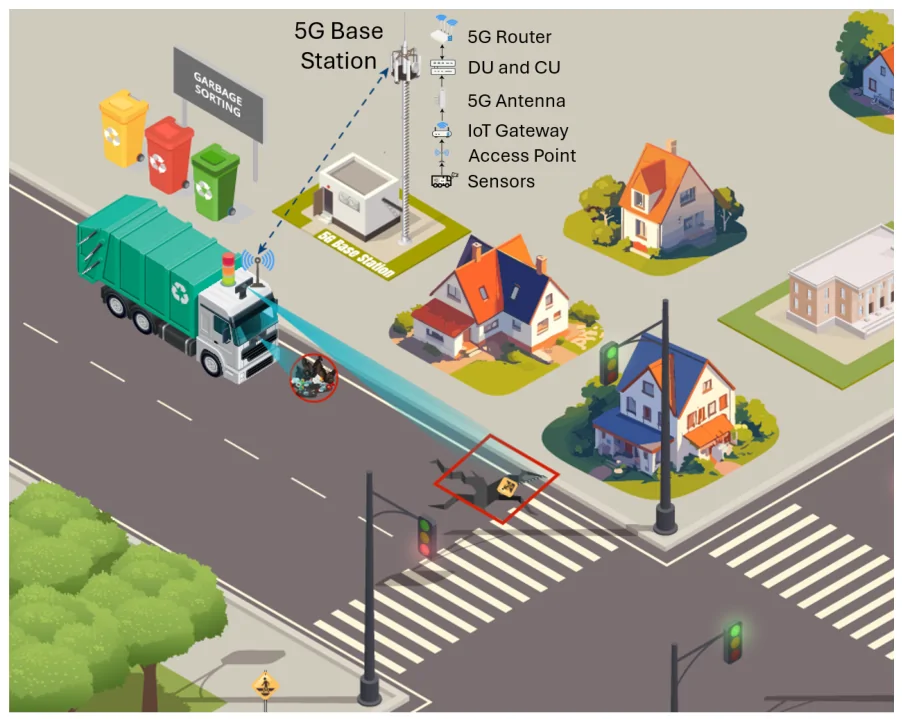
Illustration of a roadside maintenance system for a Smart City.

In [17], the authors inspected 5G network performance through an IoT Smart City application designed for automated roadside asset maintenance. In the pilot study, several waste collection service trucks were used for mobile data collection, and the collected data was sent to the cloud server using the 5G router installed on the trucks. The authors sought to understand how geographical variation, mobility and other factors affect network performance. Using the same pilot study, the authors of [18] devised a deep learning-based framework to identify roadside maintenance spots. Although both works hinge upon the same pilot study, there is a disconnect between the key objectives: (i) improving the network performance and (ii) identifying the places of maintenance in real-time. These gaps can be bridged through cognition in NDTs that jointly represent network conditions and application-level objectives, allowing applications to learn how network quality, mobility, available resources, and maintenance requirements interact and to adapt operations accordingly. The corresponding Smart City deployment scenario is shown in Figure 2.

Smart city applications rely on seamless interaction between network infrastructure, mobile IoT devices, and real-time data streams to support critical functions such as predictive maintenance, intelligent traffic control, and emergency response. However, such applications face significant challenges arising from a lack of uniform network conditions combined with a wide diversity of applications and corresponding requirements. Fluctuating network demand, interference from dense urban environments, and unpredictable mobility patterns of connected devices contribute to inconsistent data transmission and delays in decision-making. The high volume of data generated by IoT sensors strains network bandwidth and cloud processing resources, often leading to inefficiencies in data management. These challenges make optimising real-time applications challenging. These challenges can be addressed through the cognitive capabilities that enable predictive analytics to anticipate changes in network demand and spatial conditions. It is important to ensure efficient network resource utilisation and performance validation of emerging Smart City use cases before full-scale deployment. Some of the key network functions required to support Smart City applications, which adapt to dynamic network conditions in real time, are as follows:

- Fine-grained monitoring and prediction of network conditions that enable smart-city applications to adapt application quality, e.g., scheduling high-bandwidth uploads for situations with high bandwidth;
- Enabling simulation of network conditions for testing and validating new Smart City use cases before deployment, thus reducing implementation risks.

### 2.3. Remote Mining

**Figure 3:**
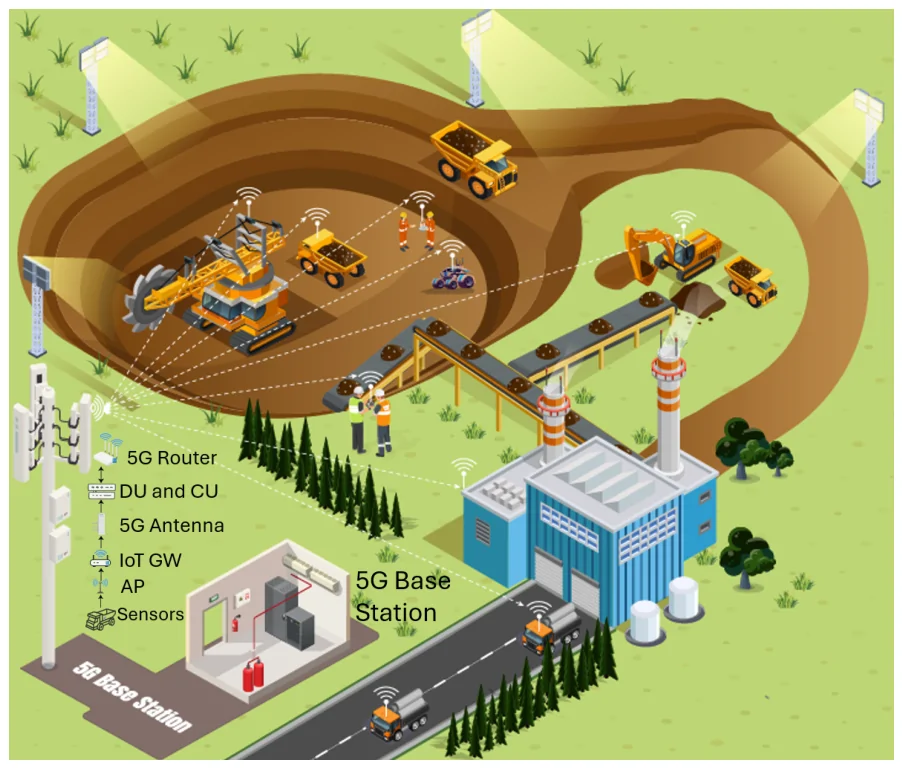
Illustration of a remote mining facility.

The authors of [19] discussed the challenges associated with remote mining and the potential of building a Digital Twin of the system for improved safety and production. Mining processes are characterised by complex operational challenges that stem from their inherently challenging environment. Their harsh operating conditions are further complicated by unpredictable weather patterns that can potentially halt surface mining activities. These multifaceted challenges demand comprehensive health and safety considerations, making the mining sector a uniquely demanding industrial domain. A representative remote mining facility is shown in Figure 3.

Continuous network monitoring and analysis of network performance, communication reliability, and environmental factors can proactively support the mining Digital Twin’s decision-making processes. This can include dynamic optimisation of communication resources, e.g., when weather conditions threaten to halt operations, it might ensure critical fleet management systems remain operational by rerouting communication channels. Further, proactive prediction of potential network failures, communication bottlenecks, or interference from environmental factors can help to mitigate occupational hazards and support the comprehensive safety protocols required in mining operations.

The above use cases highlight the need for NDTs that support application decision-making for real-time adaptation to network conditions. While recent studies have looked at Network Digital Twins that incorporate predictive and prescriptive capabilities, such as for campus networks [20] and network security monitoring [21,22], they do not explicitly consider the broader cognitive capabilities of perception, learning, reasoning, prediction, and autonomous decision-making considered in this work. These capabilities enable an NDT to interpret network conditions, learn from observations, decide on possible actions, and autonomously adapt to evolving network conditions. Existing learning-based mechanisms, including those for adaptive network security [23], can therefore be viewed as specialised cognitive components that can be integrated within a CNDT rather than replaced by it. To contextualise the subsequent discussion, Table 1 summarises the principal Digital Twin concepts considered in this review.

## 3. Digital Twin Variants: Definitions and Studies

In this section, we define and summarise the concepts, definitions, and studies of Digital Twin (DT) variants, including Cognitive Digital Twins (CDTs) and Network Digital Twins (NDTs).

### 3.1. Digital Twins

A Digital Twin (DT) is a virtual representation that serves as a real-time digital counterpart of a physical object, process, or system. It represents a bridge between the physical and digital worlds. It utilises data, machine learning, and IoT sensors to create a dynamic digital model that mimics physical counterparts ’ properties, conditions, and behaviour throughout its lifecycle. This sophisticated virtual replica enables organisations to monitor, simulate, and optimise their physical assets and processes in a risk-free virtual environment before implementing changes in the real world. The concept of Digital Twins has evolved from simple digital models to complex, dynamic representations that can predict performance, simulate scenarios, and provide actionable insights. For communication networks, a DT represents the relevant state, behaviour, and interactions of network components, including devices, links, traffic, protocols, and network resources. Across these domains, DT definitions generally agree on the use of a virtual representation that is connected to a physical system and updated using data, while differing in their emphasis on monitoring, simulation, prediction, optimisation, and autonomous decision-making.

- Across the manufacturing landscape of Industry 4.0, a DT is a comprehensive virtual representation of a physical manufacturing asset, process, or entire production system that enables real-time monitoring, predictive maintenance, and optimisation through advanced data integration and cyber–physical systems [24,25,43,44,45,46,47,48,49,50].
- In the existing literature, DT in the context of healthcare has been defined as a personalised, dynamic virtual representation of an individual patient or medical system that integrates comprehensive medical data, predictive modelling, and physiological simulations to support precision medicine, treatment optimisation, and personalised healthcare interventions [26,51,52,53,54,55,56,57,58,59].
- DT of a Smart City is a comprehensive, dynamic virtual representation of an urban environment that integrates complex urban systems, real-time data streams, and advanced simulation capabilities to support holistic urban planning, management, and sustainable development [27,60,61,62,63,64,65,66,67].
- The idea of DT has been explored in other domains such as agriculture [28,68,69,70,71,72,73,74,75,76], mining [77,78].

### 3.2. Cognitive Digital Twins

A Cognitive Digital Twin (CDT) represents a significant evolution of the traditional Digital Twin concept, incorporating advanced Artificial Intelligence, machine learning, and cognitive computing capabilities [9,29,30,31,79]. This enhanced version goes beyond basic representation and simulation, introducing human-like learning, reasoning, and autonomous decision-making capabilities. The cognitive layer allows monitoring, simulation, understanding, learning, and intelligent decision-making based on accumulated experience and real-time data analysis.

The existing literature demonstrates a rich, sometimes divergent understanding of CDT, with definitions ranging from narrow, technology-specific interpretations to broad, transformative conceptualisations.

- In the literature, a CDT is defined as an advanced digital representation of a physical system that features autonomous cognitive capabilities, enabling it to semantically link digital models across different lifecycle phases [9,31,32,79,80,81,82]. It continuously evolves in sync with the physical system, encompassing subsystems and components, and can independently execute complex activities through its enhanced cognitive support.
- The authors of [29] define an Enhanced Cognitive Twin (ECT) as an advanced digital representation that goes beyond traditional monitoring by integrating self-awareness, anomaly detection, and behavioural learning capabilities, with the crucial ability to autonomously decide and implement actions that optimise the physical twin’s performance. ECTs leverage data analytics, machine learning, and optimisation methods to transform a mere monitoring tool into an active decision-making component that improves system metrics and overall effectiveness.
- A Semantic Digital Twin (SEDIT) is defined as a virtual representation of a real-world asset that leverages semantic technologies, particularly ontologies, to create a rich, contextually aware, and standardised digital model [33,83,84,85,86,87,88,89]. By providing a shared, structured definition of domain-specific concepts, SEDIT enables a more meaningful, interoperable, and intelligent representation of physical assets, facilitating more profound understanding and more nuanced interpretation of system characteristics.
- In [30], the authors define Cognition Digital Twin (Cog-DT) as a personalised cognitive profile model that captures an individual’s unique information-taking preferences and behavioural patterns. It dynamically customises user interfaces by quantifying and tracking how a person interacts with different types, quantities, and display methods of information, ultimately aiming to reduce cognitive load and create a tailored user experience.
- The authors of [34] define a Hybrid Digital Twin (HT) to be an advanced digital representation that interconnects isolated Digital Twin models to enhance predictive capabilities by integrating diverse data sources (sensors, databases, simulations) and applying AI analytics. It enables proactive recognition and forecasting of potential suboptimal behaviours in the physical system, achieving a symbiotic relationship between interconnected models that optimise, monitor, and control system performance.

Although these definitions differ in scope, they share a common view of CDT as a Digital Twin augmented with cognitive capabilities such as learning, reasoning, prediction, and autonomous decision-making. In this work, we adopt this broader view of CDT as the conceptual foundation for Cognitive Network Digital Twins (CNDTs). A CNDT can therefore be viewed as a domain-specific CDT whose cognitive capabilities are grounded in communication network state, protocols, traffic, resources, and network control objectives.

### 3.3. Network Digital Twin (NDT)

The Zero-touch network and Service Management (ZSM) ETSI Industry Specification Group (ISG), a leading standardisation body in network management, defines the Digital Twin of a network as a virtual representation of a communications network or part of one, commonly referred to as a Network Digital Twin (NDT) [10]. This virtual representation can encompass various network components, including physical equipment, systems, processes, software environments, network elements and components, virtualised network functions, services, and network traffic patterns [90]. The modelling scope can therefore extend across multiple levels of the network stack, from network functions, links, devices, and traffic to lower-level radio and antenna components, depending on the purpose and fidelity of the twin. According to ETSI ISG specifications, an NDT must maintain comprehensive environment awareness through multiple data sources, including real-time telemetry data, network sensors, anomaly detection systems, and failure prediction mechanisms. This environmental awareness enables proactive network management and optimisation. Furthermore, the dynamic behaviour models of the NDT must continuously adapt to reflect real-time changes in both the physical network (the physical twin) and its operational environment, ensuring an accurate and up-to-date representation of the network state.

We reviewed the existing definitions in the context of Digital Twins of wireless networks in the literature, and they are listed below.

- Digital Twins for 6G can operate at multiple scales from individual entities (such as edge servers and Internet of Everything (IoE) devices), through end-to-end services (encompassing resource management, service design, and network planning), to complex multi-service systems. These Digital Twins facilitate comprehensive system analysis, design optimisation, and real-time monitoring and control of devices, ultimately enabling IoE services that reduce operational costs and resource utilisation [35].
- Network Digital Twins (NDTs) represent the next evolution in network modelling, utilising machine learning techniques to create precise, data-driven digital replicas of network systems [36,91].
- An NDT functions as a black-box model of a network system, capable of being configured through multiple parameters to simulate diverse network scenarios, including variations in topology, traffic volume, and dynamic placement policies [37].
- A Digital Twin of a Network (DTN) is a virtual representation of a communication network that continuously synchronises with real-time performance, maintenance, and health status data. Unlike physical object-focused Digital Twins, DTN specialises in abstracting network states and behaviours across universal communication networks, independent of specific applications or contexts [38].
- A 5G Digital Twin (5G DT) architecture consists of a physical 5G network and its virtual counterpart, connected through bidirectional data exchange, where the virtual twin evolves from a basic model into a comprehensive representation through AI-driven learning and continuous data updates [39,92].
- China Mobile defines a DTN as an integrated system comprising a physical network and its virtual counterpart, maintaining real-time interactive synchronisation between the two entities [40].
- An NDT creates a virtual, real-time representation of the physical network, enabling risk-free analysis, diagnosis, and emulation while facilitating informed control decisions for the network infrastructure [41].
- A Mobile Network Digital Twin (MNDT) is a virtual representation of a mobile communication network that precisely models its key components: devices, communication links, operating environment, and network applications [42].

### 3.4. Standardisation and Practical Architectural Context

Several standardisation efforts provide architectural foundations relevant to CNDT development. ISO 23247-2 defines a reference architecture for manufacturing Digital Twins through a reference model from the domain and entity perspectives together with a functional view, providing a structured basis for physical–digital representation and interaction [93]. For communication networks, ITU-T Y.3090 specifies functional and service requirements together with an architecture for Digital Twin Networks (DTNs), in which data, models, and interfaces support analysis, diagnosis, emulation, and control of the physical network [36]. ETSI ZSM further places NDTs within zero-touch network and service management by examining their integration with automated management and orchestration functions and their mapping to the ZSM architecture [10]. Collectively, these efforts establish complementary foundations for network representation, physical–digital consistency, and automated network operation. Recent practical frameworks such as B5GEMINI further demonstrate how AI/ML-enabled NDT platforms can support virtualised 5G/6G experimentation, network analysis, and intelligent control [37].

At the operational level, 3GPP and O-RAN provide analytics and control functions that can support CNDT realisation. The 3GPP Network Data Analytics Function (NWDAF), specified in TS 23.288, collects network-related information and exposes statistical and predictive analytics to network functions and applications, providing relevant analytics and contextual inputs to the CNDT data acquisition and modelling and simulation layers [94]. In O-RAN, the Non-RT RIC supports longer-timescale policy optimisation, enrichment information, and AI/ML model lifecycle functions, whereas the Near-RT RIC enables finer-grained RAN monitoring and control through the E2 interface [95,96]. These functions and interfaces align with the CNDT management, modelling and simulation, data acquisition, and synchronisation layers by supporting analytics, policy exchange, telemetry, and control. CNDTs therefore complement rather than replace these architectures, integrating their capabilities within a synchronised, context-aware, and goal-driven cognitive framework.

## 4. Cognitive Network Digital Twins (CNDTs)

Modern communication networks are growing in complexity due to the convergence of multiple technologies, such as 5G, edge computing, cloud computing, and the IoT [97,98]. These networks comprise highly heterogeneous components, including cellular base stations, fibre-optic backbones, IoT gateways, satellite links, and mobile edge computing (MEC) nodes. Furthermore, the demand for ultra-reliable low-latency communication (URLLC), massive machine-type communication (mMTC), and enhanced mobile broadband (eMBB) adds another layer of complexity. Managing such large-scale, distributed infrastructures using conventional techniques is inefficient, as they cannot dynamically adjust to evolving network conditions.

While NDTs provide valuable virtual representations of physical communication networks, their capabilities vary considerably across existing implementations. Traditional NDTs primarily focus on modelling, monitoring, simulation, and optimisation, while recent studies have incorporated learning-based optimisation, adaptive control, and autonomous decision-making into Digital Twin-based network systems [50,99,100]. These developments demonstrate that learning and adaptation are already emerging within NDT-related research. However, such capabilities are often developed for specific optimisation or control tasks, rather than being integrated as a broader cognitive capability spanning network perception, contextual knowledge, learning, reasoning, prediction, and closed-loop decision-making. This distinction motivates the need for the development of Cognitive Network Digital Twins (CNDTs).

Here we provide a formal definition of a CNDT: *A CNDT is an intelligent, self-adapting virtual representation of a communication network that continuously synchronizes with its physical counterpart while incorporating cognitive capabilities. These capabilities include perception, learning, reasoning, prediction, and autonomous decision-making, which are integrated into the modelling, simulation, and control processes of the network*.

In contrast to the predominantly monitoring- and analysis-oriented role of conventional NDTs discussed above, we define a CNDT as a twin that actively interprets network telemetry in context, maintains structured knowledge of the network and its environment, derives insights and infers latent states, updates its models through continuous learning, and autonomously adapts its behaviour or control actions. These autonomous capabilities can operate within human-defined goals, policies, and operational constraints, allowing human operators to provide guidance, monitoring, or intervention when required. These properties enable CNDTs to support closed-loop, goal-driven network operation, establishing them as a foundational element for intelligent management systems in future 6G networks.

To further delineate the progression from existing network management and Digital Twin paradigms towards CNDTs, Table 2 compares their key capabilities. Rather than implying standardised distinctions between these paradigms, the comparison highlights the increasing integration of learning, contextual reasoning, adaptation, and autonomous closed-loop decision-making.

As shown in Table 2, the defining distinction of a CNDT is not the presence of AI or automation alone, since these capabilities can also appear in SDN/SON and AI-assisted NDT implementations. Instead, a CNDT integrates perception, structured contextual knowledge, learning, reasoning, prediction, and adaptive decision-making within a synchronised, goal-driven control loop. This integrated capability set motivates the architectural taxonomy presented next.

**Figure 4:**
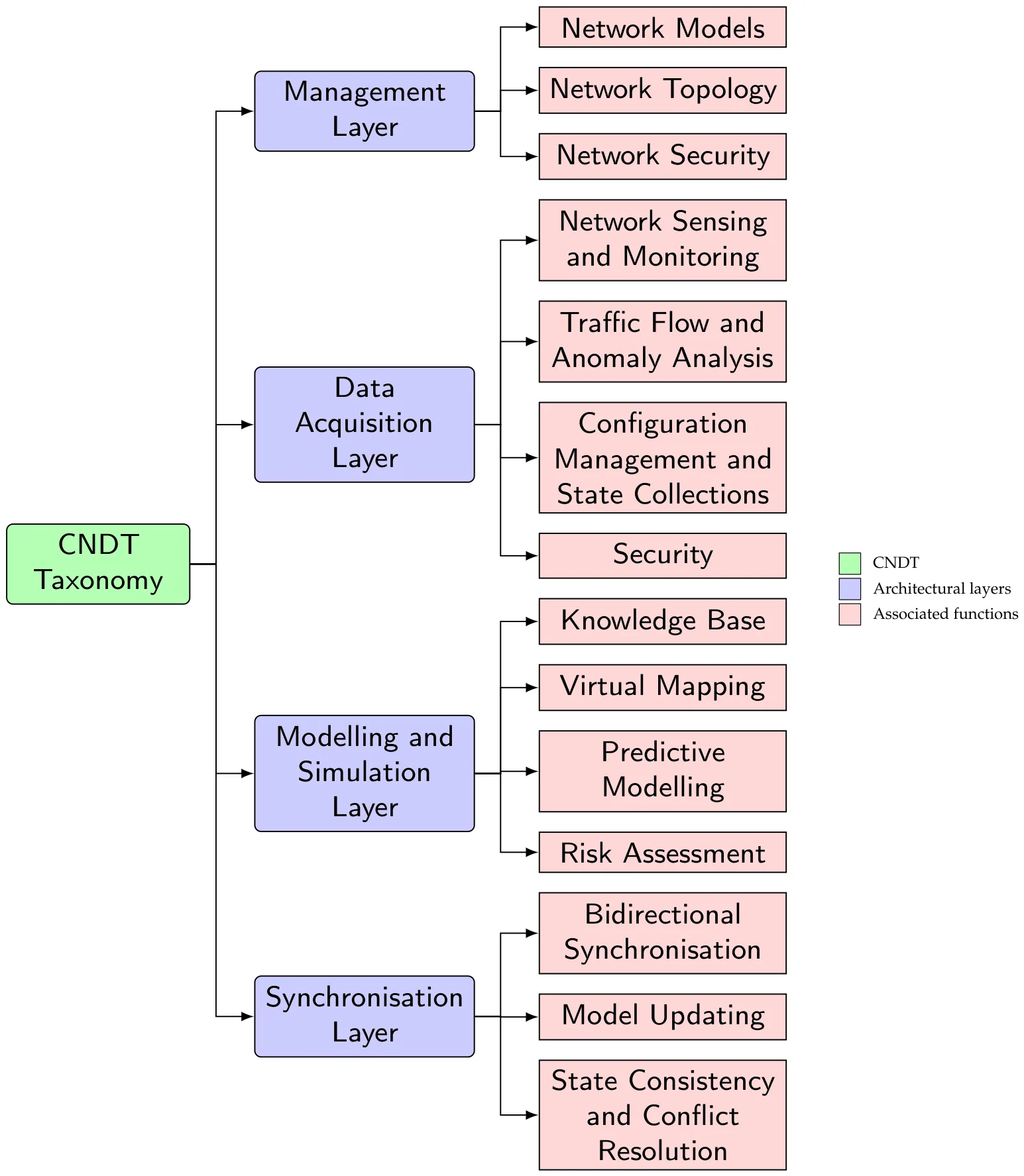
Taxonomy of Cognitive Network Digital Twins (CNDTs) spanning management, data acquisition, modelling and simulation, and synchronisation layers.

The architecture of a CNDT will be fundamentally multi-layered and intricate, comprising several critical components that enable comprehensive network representation and intelligent management. Our proposed taxonomy organises the principal architectural layers and components supporting these cognitive capabilities. In the proposed taxonomy, shown in Figure 4, perception is primarily supported by the data acquisition Layer, learning and prediction are supported by the modelling and simulation layer and continuous model updating, while reasoning and autonomous decision-making are primarily supported by the management layer. The synchronisation layer provides the continuous feedback needed to maintain these capabilities in alignment with the physical network.

**Figure 5:**
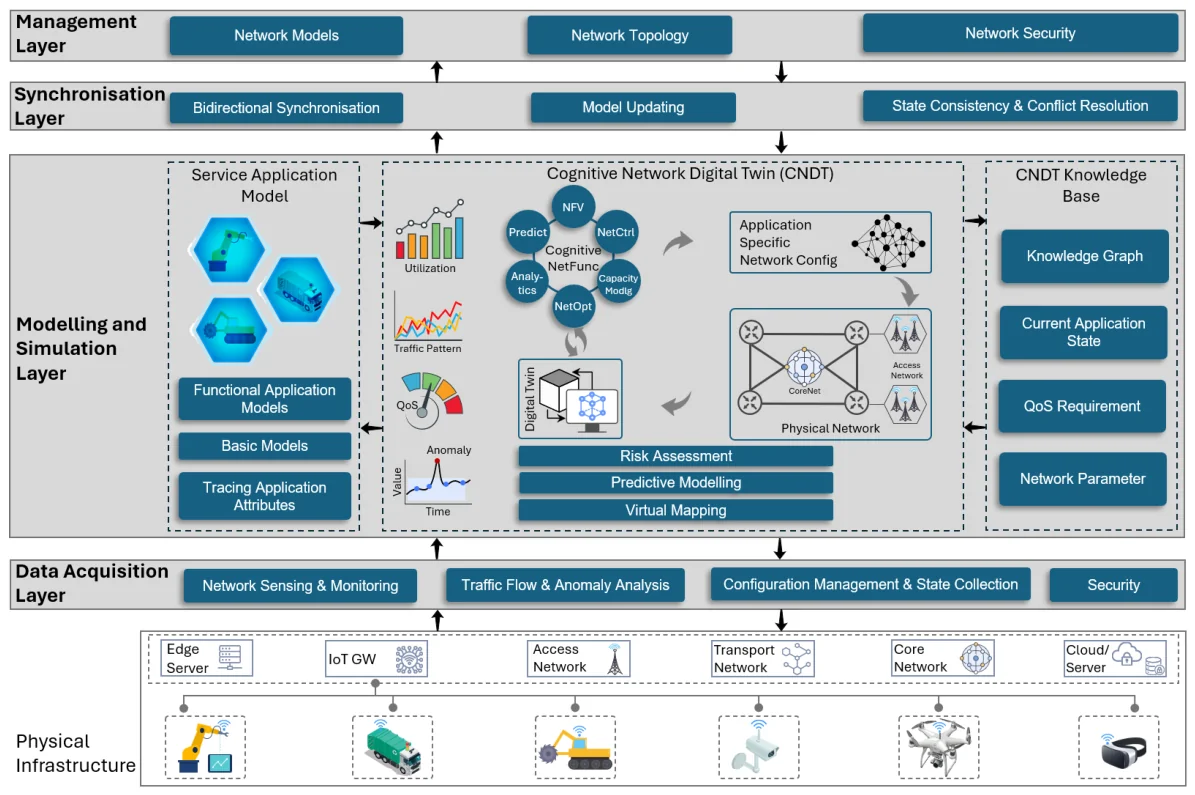
Layered architecture of a Cognitive Network Digital Twin (CNDT) comprising management, synchronisation, modelling and simulation, and data acquisition layers.

Subsequently, we now introduce a layered architecture for CNDTs, shown in Figure 5, which captures various CNDT functions into four layers. The data acquisition layer collects telemetry, traffic, configuration, and security information from the physical network, while the modelling and simulation layer transforms these data into virtual network models, predictions, and simulations. The management layer supports reasoning, decision-making, and network control, while the synchronisation layer maintains bidirectional information exchange and conveys control actions between the digital and physical networks. Together, these layers support continuous model updating and adaptive network management. Each of the layers and the functions they contain are described next.

### 4.1. Management Layer

The management layer of a CNDT provides strategic oversight and control over high-level network behaviour, structure, and security. The management layer functions as the cognitive control center of the CNDT. In the proposed architecture, the management layer is responsible for reasoning over the information provided by the lower layers and making high-level network management and control decisions. These decisions are subsequently conveyed through the synchronisation layer to the relevant network control interfaces. In the following points, we highlight the key functionalities of this layer.

- **Network Models:** This layer maintains and governs high-level network models that encapsulate the operational logic, configuration rules, service policies, and behavioural abstractions of the network. These models are critical for aligning the digital representation with real-world network goals and constraints. They act as cognitive references for anomaly detection, optimisation, and adaptation.
- **Network Topology:** Dynamic and accurate network topology management is essential for CNDT scalability and resilience. The management layer supervises the construction, validation, and updating of the logical and physical topology of the network. This includes tracking devices, links, dependencies, and policy constraints across heterogeneous infrastructure.
- **Network Security:** Given the critical role of CNDTs in autonomously managing sensitive infrastructure, network security is a central responsibility of the management layer. This includes integrating real-time threat detection, policy-based access control, and vulnerability assessments into the cognitive loop.

### 4.2. Data Acquisition Layer

The data acquisition layer forms the foundation of any NDT, acting as the critical interface between the physical network and its digital counterpart. This foundational layer involves sophisticated data collection mechanisms that gather real-time information from network elements such as physical network infrastructure sensors, performance monitoring systems, network traffic flow analysers, and configuration management databases. The key components of this layer include the following.

- **Network Sensing and Monitoring:** Modern networks generate large amounts of data. Through advanced telemetry systems and intelligent sensors, NDTs can capture everything from basic bandwidth usage to minute packet loss, jitter, and latency. This rich data enables the twin to mirror the physical network with greater accuracy.
- **Traffic Flow and Anomaly Analysis:** Beyond raw performance metrics, this sub-layer maintains vigilant oversight of traffic patterns and user behaviours. This vigilance serves a dual purpose: identifying potential bottlenecks before they impact users and detecting anomalous activities that might signal security threats. By employing sophisticated machine learning models, the system can distinguish between normal fluctuations and genuine concerns, allowing administrators to address issues proactively.
- **Configuration Management and State Collections:** The twin also maintains a comprehensive record of the network’s structural and operational state, tracking configurations, device statuses, and system parameters. When a firmware update rolls out or a policy changes, these modifications are immediately reflected in the Digital Twin, ensuring an accurate representation at all times.
- **Security:** This comprehensive sub-layer extends beyond basic metrics, including detailed logging and security analysis. The layer is integrated with intrusion detection systems to identify vulnerabilities before they can be exploited.

In [38,91,102], the authors discuss the importance of data collection from real systems in building an NDT. In particular, the authors of [38] explore the challenges associated with the data collection process. Production network data, while highly valuable for practical applications, presents significant limitations in quantity and variety. Although real data enables the development of practical deployment models, the challenge lies in collecting diverse data under various configurations, which proves unrealistic in production environments. The authors of [102] suggest a target-driven approach to collecting data from diverse sources, where the data collection parameters (type, frequency, and method) should be specifically aligned with the NDT application’s requirements. In [103], the authors present a network-wide data collection scheme based on In-band Network Telemetry (INT), inspired by the reference architecture provided in [102]. They explain that creating an accurate digital replica of the physical network requires probing paths that encompass all network links, enabling the collection of network topology data, traffic load information, and detailed port-level device statistics.

### 4.3. Modelling and Simulation Layer

The modelling and simulation layer of the CNDT is a sophisticated computational framework that transforms raw network data into meaningful, actionable representations. This layer involves high-fidelity mathematical and statistical models, machine learning-based predictive algorithms, topology mapping, network state reconstruction, and dynamic simulation capabilities to predict the network behaviour under various scenarios. The main functionalities of this layer include the following.

- **Knowledge Base:** The CNDT knowledge base serves as the semantic backbone for intelligent network representation and simulation. It encapsulates structured domain knowledge such as current network application states, Quality of Service (QoS) requirements, and network parameters (such as bandwidth, latency, and jitter). To support advanced decision-making and predictive simulation, the knowledge base can be instantiated as a knowledge graph [104], capturing relationships between entities such as nodes, services, user intents, traffic flows, and network policies.
- **Virtual Mapping:** As data flows in, this sub-layer constructs and maintains a virtual topology that precisely maps to the physical infrastructure. This virtual network exists in a computational space where administrators can observe, analyse, and interact with their network in ways previously impossible, visualising complex relationships and dependencies.
- **Predictive Modelling:** A well-designed NDT can anticipate potential failures, suggest optimisation strategies, and even refine routing paths through reinforcement learning techniques for better service quality [39]. Because network conditions can change substantially over time, the predictive models within a CNDT cannot rely solely on one-off training. Human mobility, traffic dynamics, channel conditions, and changes in network topology can introduce data and concept drift, causing previously learned models to become less accurate. Recent studies have highlighted such challenges in dynamic wireless environments [105,106] and have explored prediction mechanisms that explicitly account for temporal delays and changing spatial relationships [107]. For CNDTs, this motivates continual model updating and adaptation so that predictions remain consistent with the evolving network state.
- **Risk Assessment:** The simulation capability naturally extends to cybersecurity, enabling comprehensive risk assessment through virtual penetration testing and vulnerability scanning. By identifying security weaknesses in the simulated environment, organisations can strengthen their defenses proactively, testing defensive measures against the latest attack methodologies.

In [37], the authors designed a testbed for NDT and developed AI-based models to highlight the potential use cases of NDT in cybersecurity and energy sectors. The authors of [108] explore the main ML technologies that can enable the development of the components of the DTN architecture. In [91], the authors provide a general overview of the architecture of the NDT. They present a case study that leverages an ML-based NDT for network performance evaluation and apply it to routing optimisation in a QoS-aware use case.

### 4.4. Synchronisation Layer

This layer is a critical component ensuring continuous alignment between the physical and digital networks. This mechanism involves real-time data synchronisation protocols and bidirectional information exchange. The main tasks of this layer are as follows.

- **Bidirectional Synchronisation:** High-speed, bidirectional data streams establish a continuous feedback loop that minimises latency between real events and their representation in the twin. Unlike conventional monitoring systems that alert administrators to issues, an advanced NDT leverages this synchronisation to maintain digital–physical alignment.
- **Model Updating:** What distinguishes a CNDT is its capacity for self-improvement. The twin analyses its performance through continuous learning algorithms, refining predictions and improving accuracy over time. As it encounters new network conditions, it autonomously adapts its models to maintain relevance and becomes increasingly intelligent.
- **State Consistency and Conflict Resolution:** Maintaining state consistency between physical and virtual environments presents significant challenges, particularly in complex networks. This sublayer employs sophisticated error correction algorithms and validation techniques to prevent divergence between the real world and its digital reflection, ensuring that decisions are always based on accurate information.

The authors of [109] propose a solution from a data perspective for modelling traffic data within the Digital Twin of the network (defined as DTN). This study sets up the network analysis model within the DTN layer as the target. They propose a methodology that can be quickly trained with the real-time collected data to fit this model.

Maintaining this alignment is not free. Every update consumes bandwidth, processing and energy, and in a CNDT that spans edge and cloud resources, this cost grows with the number of monitored elements and the required freshness. The resulting trade-off between synchronisation overhead and state freshness is quantified by the KPIs in Section 4.5 and discussed as an open challenge in Section 5.

### 4.5. Key Performance Indicators for CNDTs

The cognitive capabilities introduced above are only useful if their effect on network operation can be measured. Conventional NDT evaluation focuses on the fidelity of the twin, such as the error between predicted and measured delay. A CNDT additionally closes the loop from perception to action, so its evaluation must also capture how quickly, how accurately, and at what cost it reasons and acts.

*Closed-loop decision latency* is the time from a change in the physical network to the enforcement of the corresponding control action. For URLLC services, ITU-R specifies a user-plane latency target of 1 ms [110], which bounds the loops that can act on individual transmissions. The O-RAN architecture separates control loops into real-time (below 10 ms), near-real-time (10 ms to 1 s) and non-real-time (above 1 s) classes [111], which provides a reference for classifying CNDT functions by their latency requirements.

*State synchronisation overhead* measures the resources consumed to keep the twin aligned with the physical network. It includes the telemetry and control signalling rate, the share of link capacity this signalling occupies, and the computation and energy spent ingesting and processing updates.

*State freshness* and *state divergence* capture how well the twin reflects the network at the moment a decision is made. Freshness can be quantified by the Age of Information (AoI), defined as the time elapsed since the generation of the most recent update received by the twin [112]. Divergence measures the error between the twin’s state estimate and the measured physical state, for example, the normalised error in link utilisation or queue length. Both trade off directly against synchronisation overhead.

*Prediction accuracy* under dynamic load evaluates the modelling and simulation layer across operating regimes rather than as a single average. Error metrics such as the mean absolute percentage error should be reported separately for low, nominal and peak load and during transitions such as traffic surges, handover bursts or topology changes. A related indicator, model adaptation time, is the time the twin takes to return to its nominal prediction accuracy after a distribution shift. It reflects the effectiveness of continual model updating under data and concept drift.

*Decision quality* evaluates the outcome of autonomous actions, for example, the reduction in service level agreement (SLA) violations, the utility achieved relative to an offline optimum, or the proportion of actions that are rolled back. Finally, in line with the Industry 5.0 perspective, the *human intervention rate* records how often operators override or correct CNDT decisions. It indicates both the trustworthiness of the twin and the balance between autonomy and human oversight.

The relative importance of these KPIs depends on the application. In the smart manufacturing use case (Section 2.1), robotic coordination and AR/VR-assisted remote access make closed-loop latency and prediction accuracy during load transitions the leading concerns. In the Smart City use case (Section 2.2), device mobility makes state freshness and model adaptation time critical. In remote mining (Section 2.3), where weather and harsh conditions can disrupt links, decision quality under degraded connectivity and synchronisation overhead on constrained backhaul become decisive. Since the KPIs are interdependent, CNDT evaluations should report them jointly rather than optimising any single indicator.

## 5. Open Research Challenges

CNDTs integrate AI/ML and cognitive computing capabilities to enable proactive decision-making, predictive analytics, and autonomous network management. Hence, they should dynamically adjust network configurations, anticipate failures, and optimise resource utilisation in real-time. Here we discuss the key open challenges that will drive future research in the field of CNDTs.

### 5.1. Managing Knowledge

Managing knowledge is a crucial aspect of CNDT development. For a CNDT to function effectively, it must represent, acquire, and update knowledge efficiently. Formal knowledge representation techniques are needed to structure the information CNDTs learn from experience, enabling efficient reasoning and decision-making. Knowledge acquisition mechanisms must also be improved to allow CNDTs to extract valuable insights from diverse data sources, such as network telemetry, logs, and user interactions. Moreover, knowledge updating is a significant challenge, as networks are constantly evolving, leading to potential data drift and outdated models. Ensuring NDTs can self-update and recalibrate their knowledge base without manual intervention is a critical area requiring further investigation.

For Industry 5.0 applications, human expertise and interaction should also be considered as part of the knowledge available to CNDTs. Human operators may provide operational objectives, feedback, or domain-specific knowledge, while CNDTs can use this information together with network telemetry to support autonomous decision-making. Determining how such human input can be incorporated while retaining effective autonomous operation remains an open research challenge.

### 5.2. Data Integration and Modelling

Data integration and modelling form another critical research challenge in CNDTs. Networks generate vast amounts of data from multiple sources, including sensors, logs, performance metrics, and user interactions. However, integrating these diverse data streams into a unified digital representation remains complex due to inconsistencies in formats, structures, and semantics. Existing approaches lack standardised methods for semantic integration, making it difficult for CNDTs to interpret and combine data from heterogeneous systems. Furthermore, real-time, low-latency data processing is essential to ensure that CNDTs can adapt to rapid changes in network conditions. Developing scalable, interoperable data integration frameworks is necessary to enhance the accuracy and responsiveness of CNDTs.

### 5.3. Integrating CNDT Models

Integrating CNDT models across different stakeholders and network environments presents another challenge. Organisations such as telecom operators, cloud providers, and enterprises may develop CNDTs with distinct architectures, optimisation criteria, and data models. Ensuring seamless interoperability among these diverse CNDT instances enables cross-domain collaboration and unified network management [113]. Standardisation efforts must focus on developing standard protocols, data formats, and orchestration frameworks that facilitate the integration of CNDT models across heterogeneous environments. Real-world deployment scenarios must be explored to identify practical challenges and refine integration strategies accordingly.

### 5.4. Lifecycle Management

Lifecycle management of CNDTs remains an open research area that requires significant attention. CNDTs must continuously evolve to keep pace with changes in the physical network, necessitating robust methods for real-time updates and adaptation. A major challenge is ensuring that CNDTs remain synchronised with real-world network conditions, especially as traffic patterns, device behaviours, and external influences shift over time. Effective lifecycle management strategies should address data drift and concept evolution, allowing CNDTs to self-correct and maintain relevance. Moreover, as CNDTs gain more control over network operations, they become potential targets for cyber threats. Implementing security frameworks that protect against adversarial attacks while maintaining operational integrity is crucial for the long-term resilience of CNDTs.

### 5.5. Placement and Implementation Strategies

Another key research gap pertains to the strategic placement and architectural design of CNDTs. Networks vary widely in scale, topology, and operational constraints, necessitating different approaches to CNDT deployment. While centralised CNDTs may be suitable for stable, controlled environments, large-scale, distributed networks require decentralised and federated CNDT architectures. Furthermore, networks associated with mobile systems, such as vehicular networks and UAV communication systems, introduce new challenges in CNDT mobility and real-time migration, which have not been adequately addressed in existing studies.

A significant research gap is determining whether CNDTs should follow a centralised or distributed deployment model based on network characteristics, scale, and operational requirements.

#### 5.5.1. Centralised CNDTs

These can be hosted in centralised cloud environments or core network infrastructures, offering high computational power and global network visibility. However, centralised architectures introduce latency issues, as real-time data from geographically dispersed network components must be transmitted to a remote processing centre. Centralised CNDTs can have scalability limitations, making accommodating modern networks’ dynamic and large-scale nature challenging, particularly in 5G/6G network environments.

#### 5.5.2. Distributed CNDTs

This involves deploying multiple CNDT instances across edge and fog nodes, providing localised intelligence and reducing latency. However, distributed architectures introduce synchronisation challenges, as different CNDT instances need to maintain a consistent and coherent network representation.

The primary factors that determine the CNDT placement strategy are the Quality of Experience (QoE) characteristics of the application [114]. This includes application Quality of Service (QoS) requirements, including throughput, latency, jitter, etc.; network traffic characteristics; and application characteristics, such as the level of mobility. For instance, the use cases outlined earlier exhibit distinct performance requirements. While smart manufacturing applications are likely to be highly time-sensitive, with strict latency and jitter requirements, Smart City applications are likely to be characterised by mobility. Key research challenges here relate to designing placement and implementation strategies that ensure that CNDTs can adapt to application QoE requirements. As indicated above, this may imply different strategies based on application domains.

Current research lacks a comprehensive comparison and decision framework that helps determine the most suitable CNDT placement strategy based on network topology, computational needs, and application-specific requirements.

### 5.6. Predictive Maintenance and Fault Management

In mission-critical applications such as industrial automation [115], smart grids [116], and remote healthcare [117], network failures can lead to severe disruptions, financial losses, or even safety hazards. Traditional fault management relies on reactive maintenance, where issues are only addressed after they occur. This reactive approach is inefficient and costly, often requiring extensive manual intervention and troubleshooting. Furthermore, unexpected failures can lead to prolonged downtimes, negatively impacting service quality and business operations. CNDTs introduce predictive maintenance capabilities [118], where AI models analyse historical and real-time network data to detect early signs of degradation. By predicting failures before they occur, CNDTs can trigger proactive mitigation strategies, such as rerouting traffic, dynamically switching to backup network paths, or preemptively alerting maintenance teams. This proactive approach reduces downtime, minimises repair costs, and enhances overall network resilience.

### 5.7. Telemetry Overhead and Edge–Cloud Synchronisation

A monitoring-oriented NDT can often tolerate coarse, periodic telemetry, whereas a CNDT may require finer-grained and more frequent telemetry to support its cognitive functions. Learning and drift detection require representative samples across operating regimes, reasoning over the knowledge base requires up-to-date context, and closed-loop decisions require a fresh view of the state being acted upon. Network-wide approaches such as ’in-band network telemetry’ make fine-grained collection possible, but probing every link and collecting port-level statistics at high frequency adds traffic to the very network being managed.

This overhead is most acute at the boundary between edge and cloud. Edge sites close to base stations, IoT gateways, and MEC nodes generate most of the raw telemetry, whereas the global network view, long-term knowledge and computationally intensive models typically reside in the cloud or core. The backhaul links between them are shared with user traffic and, in settings such as remote mining, may be constrained or intermittent.

*Data compression and reduction:* Several complementary techniques can reduce the volume of data crossing the edge–cloud boundary. Sketch-based measurement summarises per-flow traffic statistics in compact data structures that are updated at line rate and can be compressed to fit the bandwidth available for export [119]. Edge-side feature extraction forwards derived quantities, such as aggregated statistics, anomaly scores or learned embeddings, instead of raw counters and logs. Model-based (residual) reporting exploits the twin’s own predictive models. If the edge and cloud instances share the same model, the edge transmits only when the measured state deviates from the prediction beyond a tolerance, so predictable behaviour costs little signalling. More broadly, goal-oriented and semantic communication suggests transmitting only the information relevant to the decision the twin must make, rather than reproducing the data exactly [120]. Each approach trades fidelity for bandwidth, and the acceptable loss depends on the downstream cognitive function. A capacity-planning model may tolerate aggregated data, whereas an anomaly detector may lose the very outliers it is designed to find.

*Event-driven updates:* Periodic polling sends updates whether or not the state has changed, which wastes resources during stable periods and lags behind rapid change. Event-driven synchronisation instead triggers updates on state changes, threshold crossings or predicted deviations, and freshness metrics derived from the AoI provide a principled basis for designing such policies. Recent work schedules Digital Twin updates to minimise a weighted sum of the Age of Changed Information and the update cost, yielding threshold-based policies for twins hosted in MEC environments [121]. Other work uses one-step state predictions to decide which physical objects to synchronise first [122] or applies AoI and error-aware scheduling within a cloud–edge collaborative twin [123]. Extending these schemes from individual objects to network-wide CNDTs, in which many interdependent elements share the same telemetry channels, remains a possibility. Event-driven policies also carry a specific risk, where the gradual drift that never crosses a trigger threshold can accumulate without notice, so they need to be combined with periodic consistency checks.

*Edge–cloud consistency:* Placing twin instances on edge servers close to their physical counterparts reduces synchronisation latency [124], and hierarchical designs combine such edge twins with a global twin in the cloud. This raises the question of which consistency model the instances should follow. Strong consistency simplifies reasoning but adds coordination delay to every update, whereas eventual consistency lowers delay but allows edge and cloud instances to act on conflicting views of the network. Open research enquiries therefore include (i) adapting compression ratios and update frequencies to the KPI targets of each application; (ii) defining consistency guarantees for distributed CNDT instances, including the placement challenges; and (iii) determining the amount of telemetry reduction the cognitive models can tolerate before decision quality degrades.

### 5.8. Security of Autonomous Cognitive Control Loops

Security threats to Digital Twins in general, including attacks on data, models and the communication between physical and virtual entities, have been surveyed extensively [125]. CNDTs raise a greater concern because they not only observe the network but also act on it. An adversary who can influence what a CNDT perceives, knows, or learns can therefore steer real control decisions through the cognitive loop, and the twin’s autonomy may propagate a single manipulation across the network faster than an operator can react. Adversarial machine learning provides the general framework for these threats [126]. Below, we outline how they map onto the CNDT layers introduced in Section 4.

*Data poisoning in the data acquisition layer:* Compromised devices, spoofed telemetry, or manipulated logs can inject falsified measurements into the twin. Because CNDT models are updated continually (Section 4.4), poisoned data can enter training as well as inference, gradually shifting what the twin regards as normal behaviour. Continual learning also makes it difficult to separate genuine concept drift from deliberate poisoning, since both appear as a change in the data distribution.

*Semantic poisoning of the knowledge base:* When the knowledge base is instantiated as a knowledge graph (Section 4.3), an attacker may insert, delete, or alter facts, such as a false dependency between services, an incorrect QoS requirement, or a forged policy. Each change may be small and plausible in isolation, yet it alters the context used for reasoning. Attacks of this kind have been demonstrated against knowledge graph-embedding models, where adding or deleting a small number of carefully chosen facts manipulates the plausibility of targeted facts [127].

*Evasion of learned detectors:* The anomaly and intrusion detection functions in the data acquisition and management layers are typically ML-based, and adversarially crafted traffic can evade them [128]. In the radio domain, small perturbations of received signals can likewise mislead learning-based spectrum sensing and signal classification [129].

*Manipulation of learning-based controllers:* Reinforcement learning agents used for routing, resource allocation or admission control can be misled by perturbed observations, manipulated rewards, or poisoned experience [130]. An example from intelligent transportation shows that colluding vehicles reporting falsified state information can steer a deep reinforcement learning traffic signal controller to their own advantage [131]. The same pattern applies to network devices or users that misreport their state to a CNDT.

*Mitigation directions:* Several properties of CNDTs can be turned into defences. First, the twin’s own models offer a plausibility reference: telemetry that contradicts physical constraints or the predictions of independent models can be flagged before it enters learning or reasoning. Second, provenance tracking and authentication of knowledge base updates make semantic changes auditable and reversible. Third, the twin can act as a sandbox, simulating the consequences of a proposed action before it is enforced on the physical network. Fourth, safety shields can restrict learned policies to actions that satisfy formally specified constraints [132]. Finally, consistent with the Industry 5.0 perspective, high-impact or low-confidence actions can require operator approval, which links the human intervention rate KPI (Section 4.5) to security. Remaining challenges include distinguishing poisoning from legitimate drift during continual learning, certifying the robustness of cognitive models used in closed loops, and achieving both within the latency budgets discussed in Section 5.7.

### 5.9. Computational Overhead of Cognitive Functions Under Real-Time Constraints

The cognitive functions that distinguish a CNDT from a conventional NDT also add computation to every pass through the synchronisation loop. For URLLC services, ITU-R sets a user-plane latency target of 1 ms [110], which leaves little or no room for model inference inside a per-transmission control loop. URLLC is also defined by its reliability, which makes the tail of the latency distribution, rather than its mean, the relevant measure [133]. Inference time varies with input size, hardware contention and model complexity, so a model that is fast on average may still miss its deadline in the cases that matter most.

*The cost also scales with the twin’s scope:* Graph neural network models such as RouteNet were developed as a lower-cost alternative to packet-level simulation for estimating network performance [134], and they make ML-based NDTs practical. However, a CNDT that evaluates many candidate actions, simulates what-if scenarios, or retrains its models online multiplies this per-evaluation cost. Continuous model updating (Section 4.4) also competes with inference for the same computation resources unless the two are separated.

*Timescale separation:* A practical response is to assign each cognitive function to a control loop whose timescale matches its computational cost. The O-RAN architecture already distinguishes real-time loops (below 10 ms), near-real-time loops (10 ms to 1 s) hosted in the near-real-time RAN Intelligent Controller, and non-real-time loops (above 1 s) hosted in the non-real-time controller [111]. In a CNDT, the sub-millisecond loops that protect URLLC traffic would execute lightweight, pre-computed policies close to the physical network rather than query the full twin. Near-real-time loops can host predictive models and fast reasoning for resource allocation and mobility management. Non-real-time loops would host computationally heavy functions, such as model retraining, what-if simulation, knowledge base maintenance and policy synthesis, whose outputs are pushed down as updated policies or models. In this arrangement, the full cognitive twin shapes the behaviour of the fast loops without sitting on their critical path.

*Reducing inference cost:* Within each loop, edge intelligence techniques can reduce inference latency. These include model compression through pruning, quantisation or knowledge distillation, partitioning a model between devices and edge servers and early-exit architectures that stop computation once a sufficiently confident result is reached [135]. Predictive operation offers a further route: if the twin anticipates a load surge or failure, it can compute and stage the corresponding action in advance, so that only a lookup remains at decision time.

Remaining challenges include (i) jointly allocating communication and computation resources so that the end-to-end loop meets its deadline with the required reliability; (ii) characterising the accuracy–latency trade-off of compressed cognitive models in network control, where a faster but less accurate model may produce worse decisions than a slower one; and (iii) deciding which functions must run at the edge, which links this challenge to the placement strategies in Section 5.5.

## 6. Conclusions

While Network Digital Twins have grown in prominence for enabling fine-grained real-time network monitoring, existing approaches have primarily focused on monitoring and optimising the network stack, with relatively limited attention to application adaptability under varying network conditions. In this paper, we explored the necessity and role of cognition in Network Digital Twins that can enable applications to adapt to changing network conditions dynamically. Based on motivating use-cases of emerging data-intensive applications in industrial domains, we identified key network functions required to enable applications to adapt to varying network conditions in real time. Our review of the current state of the art in NDTs highlighted a lack of cognitive capabilities required to support application network adaptation. We introduced Cognitive Network Digital Twins (CNDTs) as an approach to address these gaps and proposed a CNDT architecture that identifies and organises key CNDT functions.

We have outlined a comprehensive CNDT research agenda with a discussion on the key research topics. To the best of our knowledge, this work represents an early contribution that explicitly positions CNDTs as a distinct research focus in addressing the networking requirements of emerging data-intensive applications. Here, we note that while this paper lays out a research agenda identifying research directions for CNDTs, each of the topics outlined warrant individual research focus.

## Figures and Tables

**Table 1:** Summary of Digital Twin concepts.

Category	Brief Definition	Key Characteristics	References
Digital Twin	A virtual representation of a physical object, system, or process that updates dynamically with real-time data.	Bridges physical and digital worlds Uses IoT, AI, and ML for modelling Simulates and optimises processes Applied in manufacturing, healthcare, and smart cities	[24,25,26,27,28]
Cognitive Digital Twin (CDT)	A Digital Twin enhanced with AI, cognitive computing, and learning capabilities for intelligent decision-making.	Self-learning and adaptive Integrates AI and machine learning Predicts, optimises, and automates tasks Variants include Enhanced, Semantic, and Hybrid Digital Twins	[29,30,31,32,33,34]
Network Digital Twin (NDT)	A real-time virtual model of a communication network that mirrors network performance, traffic, and operations.	Models network components and behaviours Enables simulation, fault detection, and optimisation Applied in 5G/6G, IoT, and cloud networks Uses AI-driven analytics	[10,35,36,37,38,39,40,41,42]

**Table 2:** Evolution in capability from conventional network management to Cognitive Network Digital Twins.

Capability	SDN/SON-Based Management [7,101]	Conventional NDT [35,36,91]	AI-Assisted NDT [37,39]	CNDT
Primary role	Programmable and automated network management	Network representation, analysis, and simulation	Learning-assisted prediction and optimisation	Cognitive, goal-driven network management
Network-state representation	Operational state and controller views	Synchronised virtual representation of network state	Virtual representation augmented by learned models	Context-aware and dynamically evolving network representation
Learning and prediction	Function-specific; ML increasingly integrated	Optional and implementation-dependent	Central to selected prediction or optimisation tasks	Integrated with reasoning and continual adaptation
Semantic/contextual reasoning	Not intrinsic	Generally limited	Not necessarily explicit; task-specific where implemented	Explicit structured knowledge and contextual reasoning
Autonomy level	Policy- or rule-driven automation	Decision support and control; autonomy is implementation-dependent	Autonomous operation within selected learned or optimised tasks	Goal- and constraint-aware autonomous decision-making
Closed-loop operation	Predefined or policy-driven control loops	Supports closed-loop automation; scope is implementation-dependent	Learning-enabled control for selected tasks	Integrated perception-to-action cognitive loop
Closed-loop latency/timescale	Function- and implementation-dependent	Application- and implementation-dependent	Task-dependent	Multi-timescale and latency-aware
Adaptation scope	Configuration and parameter optimisation	Twin-state and model adaptation	Adaptation of selected models or policies	Joint adaptation of state, models, knowledge, and control
5G/6G KPI and service-objective	KPI- and policy-driven operation	Performance representation and analysis	Optimisation of selected KPIs or objectives	Context- and goal-aware coordination across multiple KPIs and service objectives

## Data Availability

No new data were created or analyzed in this study. Data sharing is not applicable to this article.
